# The experience of participating in an internet-based cognitive behavioral therapy program among patients with cardiovascular disease and depression: a qualitative interview study

**DOI:** 10.1186/s12888-022-03939-7

**Published:** 2022-04-25

**Authors:** Mats Westas, Ghassan Mourad, Gerhard Andersson, Margit Neher, Johan Lundgren, Peter Johansson

**Affiliations:** 1 Department of Health, Medicine and Caring Sciences, Linköping University, Linköping, Sweden; 2 Department of Behavioural Sciences and Learning, Department of Biomedical and Clinical Sciences, Linköping University, Linköping, Sweden; 3 Department of Clinical Neuroscience, Karolinska Institute, Stockholm, Sweden; 4 Department of Rehabilitation, School of Health and Welfare, Jönköping University, Jönköping, Sweden

**Keywords:** Internet-based CBT, Cardiovascular disease, Depression, Qualitative research, Thematic analysis, Patient experiences

## Abstract

**Background:**

Depression in conjunction with cardiovascular disease (CVD) is associated with worsening in CVD, higher mortality, and poorer quality of life. Despite the poor outcomes there is a treatment gap of depression in CVD patients. Recently we found that an Internet-based cognitive behavioral therapy (iCBT) tailored for CVD patients led to reduced symptoms of depression. However, we still have little knowledge about CVD patients’ experiences of working with iCBT. The aim of this study was therefore to explore CVD patients’ experiences of engaging in a tailored iCBT program.

**Methods:**

A qualitative interview study using inductive thematic analysis. Data was obtained from 20 patients with CVD and depressive symptoms who had participated in a randomized controlled trial (RCT) evaluating the impact of a nine-week iCBT program on depression.

**Results:**

Three main themes emerged: (1) Taking control of the disease, (2) Not just a walk in the park, and (3) Feeling a personal engagement with the iCBT program. The first theme included comments that the tailored program gave the patients a feeling of being active in the treatment process and helped them achieve changes in thoughts and behaviors necessary to take control of their CVD. The second theme showed that patients also experienced the program as demanding and emotionally challenging. However, it was viewed as helpful to challenge negative thinking about living with CVD and to change depressive thoughts. In the third theme patients reported that the structure inherent in the program, in the form of organizing their own health and the scheduled feedback from the therapist created a feeling of being seen as an individual. The feeling of being acknowledged as a person also made it easier to continuously work with the changes necessary to improve their health.

**Conclusions:**

Engaging in an iCBT program tailored for patients with CVD and depression was by the patients perceived as helpful in the treatment of depression. They experienced positive changes in emotions, thoughts, and behaviors which a result of learning to take control of their CVD, being confirmed and getting support. The patients considered working with the iCBT program as demanding and emotionally challenging, but necessary to achieve changes in emotions, thoughts, and behaviors.

**Supplementary Information:**

The online version contains supplementary material available at 10.1186/s12888-022-03939-7.

## Background

Studies suggest that among patients with cardiovascular disease 20–40% have depressive symptoms, which is significantly higher than the rate of depression in the general population [1, 2]. Depression in patients with CVD is associated with several negative effects, such as lower health-related quality of life [1], increased risk of morbidity and all-cause mortality [3, 4]. Thus, depression is a common and serious problem in patients with cardiovascular disease. This has also been highlighted by the European Society of Cardiology Guidelines [5] and the American Heart Association [6], .both of whom suggest that it is important to detect and treat depression in CVD patients.

There are, however, several challenges in the treatment of depression in cardiovascular disease. One challenge is that the effects of pharmacological treatment are generally small [1] and can also increase the risk of interactions with other medications and side effects [7]. One alternative to pharmacological treatment is psychological interventions such as cognitive behavioral therapy (CBT). Although a recognized method in the treatment of depression in cardiovascular patients [8, 9], CBT brings another challenge. Traditional face-to-face CBT suffers from limited access to psychotherapists and the high cost of the treatment [10]. A possible solution could be internet-based CBT (iCBT) which, compared to face-to-face CBT, has been found to be equally effective and also a cost-efficient treatment option for depression in other populations [11, 12]. iCBT can be delivered in an unguided or guided format with feedback on weekly homework assignments. Most research suggests that the guided format is more effective and less associated with dropout [13]. Moreover, iCBT can be delivered to patients in their home by healthcare providers with only brief introductory training in CBT, allowing more patients to have access to the treatment [14].

.Furthermore, studies report that patients with chronic somatic diseases who work in generic iCBT programs have difficulties to identify themselves when defining problems and in goal settings [15, 16]. In another iCBT study, CVD patients expressed that an improvement of the program was to include the possibility to ask medical questions [17]. Thus, to maximize the potential health benefits of iCBT in patients with chronic somatic diseases, suggestions have therefore been made that these programs should be adapted to the specific chronic somatic condition [15, 16, 18], such as CVD in our study.

Our research team recently published a randomized controlled (RCT) trial in which an iCBT program was tested in patients with CVD and depression. The program was tailored for this patient group and guided by nurses with brief introductory training in CBT and in collaboration with a clinical psychologist. One of the nurses was a specialist psychological nurse (JL) [19]. The results showed that the iCBT program had significant and moderate effects on depression. This demonstrates that it is possible to treat depression in patients with CVD using tailored and guided iCBT. However, the study did not provide any in-depth information regarding depressed cardiovascular patients’ experiences of taking part in the tailored and guided iCBT program.

A central aspect of CBT is changing the patterns of emotions, thoughts, and behaviors in order to decrease the symptoms of depression [20]. It is therefore important to understand how the iCBT program influenced emotions, thoughts, and behaviors in patients with cardiovascular disease. To our knowledge there are only two studies exploring patients with CVD and their experiences of participating in guided iCBT [17, 21]. In a qualitative study [21], heart failure patients reported that information about heart failure and depression was a helpful way to learn about self-care and strategies and to cope with their own health. In another study, based on both qualitative and quantitative data, patients with a recent myocardial infarction reported both positive and negative experiences of participating in an RCT. For example, some patients valued the content of the program and the therapist contact while others did not [17]. However, these two studies focused more on the experience of participation and the structure of the iCBT program and did not specifically explore the how engaging in an iCBT program influenced to changes in emotions, thoughts, and behaviors.

## Method

### Design

We used a qualitative semi-structured interview study design when interviewing patients who had participated in an iCBT intervention. One-to-one interviews with open-ended questions were conducted and subsequently analyzed using inductive thematic analysis [22].

### The intervention

The nine-week iCBT program tailored for CVD patients (heart failure, atrial fibrillation, and coronary artery disease) consisted of 7 modules with weekly work and assignments according to the treatment steps in CBT including goal setting, disease-oriented psychoeducation, problem solving and behavioral activation. The present iCBT program was developed from a previous iCBT program targeting depression in patients with heart failure [23, 24]. Each of the 7 modules consists of text, figures, videos and homework assignments adopted to fit the context of CVD. At the end of each week, patients received feedback on the completed homework assignment by a nurse therapist, who had received a short course in CBT. The nurse therapists had also experience in cardiac care and were able to answer CVD-related questions. Using nurse therapist with experience in cardiac care has been shown to be effective in a recent study [25]. The feedback was personalized and focused on confirmation, encouragement and reflection and were only provided through written messages using a secure message function within the study platform [26]. No face-to-face contact was made with the patients during the trial and the patients were connected to the same therapist throughout the treatment. If the weekly assignment was not completed, patients received a total of three reminders during a consecutive period of 2 weeks. A total of 60% completed all 7 modules and 82% completed more than half of the modules. The mean time for feedback by the nurse therapist was 13 min/patients and week. The nurse therapist had the opportunity to consult a psychologist throughout the treatment period. A brief overview of the treatment and the 7 modules in the iCBT program is illustrated in Additional file 1. Full details of the program are published elsewhere [19].

### Subjects

The patients in the present study were recruited from a RCT evaluating the effect of iCBT on CVD patients with depression, and who were randomized to the iCBT arm (*n* = 72) [19]. In that study the inclusion criteria were being 18 years or older, receiving CVD treatment according to the current guidelines, having stable CVD with no hospitalization related to CVD in the past 4 weeks, and having at least mild depressive symptoms. Patients also needed to have regular access to a computer with an internet connection and a mobile phone. To the present interview study, we aimed to include patients with maximal variation regarding age, gender, education, the number of iCBT treatment modules performed (Additional file 1) and the type of CVD diagnosis (Table 1). Thirty-five of the seventy-two patients in the iCBT treatment group were invited by email to participate in the study and 20 (57%) of them agreed and were interviewed. Those who did not respond to the invitation, did not give any reasons for not participating, but did not differ in characteristics from the interview group regarding age, gender, education, number of treatment modules performed, type of CVD diagnosis, or depression severity at baseline.

**Table 1:** Characteristics of the participants (*n* = 20)

Characteristics	Frequency (***n*** = 20)	%
Gender		
Male	11	55
Age		
Mean year (range)	62 (34-79)	
Marital Status		
Living with partner	17	85
Living alone	3	15
Education		
Elementary	2	10
Upper secondary/high school	7	35
University	11	55
Occupation		
Working	12	60
Retired	8	40
Clinical		
Heart failure	1	5
Atrial fibrillation	11	55
Coronary artery disease/ Myocardial infarction /angina	8	40
Number of performed modules		
1-3	0	0
4-5	2	10
6-7	18	90

### Data collection

Interviews were conducted between 1 and 6 months after iCBT completion. Patients were interviewed by telephone between November 2017 and April 2018 and the interviews had a mean duration of 28 min (range 15–49 min). All interviews were conducted by the first author (MW), a PhD student and registered nurse specializing in primary healthcare and experienced in conducting health assessments by telephone. At the time of the interviews MW was working part-time in a general practice health clinic. To ensure that the topics of interest would be addressed, an open-ended interview guide was created [27]. The interview guide contained questions to capture the experience of engaging in the iCBT program and was pilot tested and revised with the members of the research team before the first patient was interviewed. As an example, patients were asked to describe their experiences of participating in the iCBT program. The interview guide used open-ended questions to allow the patients to freely describe their experiences (Table 2). No field notes were taken during the interviews. All the data from the interviews were collected by the interviewer (MW), who had previous experience of collecting and handling interview data. All interviews were conducted in the participants´ native language. All the meaning units (quotes) in the coding schedule were translated into English for presentation.

**Table 2:** Samples of interview guide

Interview guide	
**Introduction:** **You have been in contact with the health service and been treated for your heart disease. In conjunction with this, you have also been treated for depression using our online CBT program.**	
Q1- Talk a little about your heart disease	
**You have been given the opportunity to participate in an online CBT program, the aim of which was to manage and reduce depression in conjunction with heart disease.**	
Q2- What are your experiences from your participation in this program?	
a What have the different treatment modules meant to you? b What have the homework tasks meant to you? c What has the therapist’s feedback meant to you? d What are or have been important in getting you to work on changes to your health?	
Q3- What are or have been the advantages and disadvantages of the program being delivered online rather than through meetings in person?	
a Have you felt there are any problems with the program or the treatment?	
Q4- Why did you become interested in participating in this research project?	
a What were your thoughts and what was important to you when you decided you wanted to participate in the project?	
**In the future, this or similar online programs may be introduced into the health service as part of treatment.**	
Q5- On the basis of your experiences of online CBT, what do you believe is important?	
Total number of main questions represents the overall format.	

*CBT* Cognitive Behavioral Therapy

All interviews were conducted in the participant’s native language. The interview guide is translated into English for presentation purposes

Telephone interviews were used as they fitted the design of the present study, enabling a broad sampling among patients spread over the southeast part of Sweden. Telephone interviews have also shown to reduce emotional distress since the patients are not influenced by the presence of the interviewer [28]. All interviews were performed with ethical considerations regarding qualitative interviewing [29]. The participants were informed about the interviewer’s role in the study in the invitation letter for the qualitative study. All patients did provide a written informed consent prior to taking part in the interview study. The interviewer (MW) had no previous relationship to any of the participants as a therapist or in the recruitment process for the RCT study.

### Data analysis

The interviews were uploaded and transcribed verbatim. The first author (MW) checked the transcribed interviews for accuracy prior to the analysis. Thematic analysis with an inductive and latent approach was used to explore the experience of how engagement in an iCBT program could influence possible changes in emotions, thoughts, and behaviors. The inductive approach allows to generate themes from patterns in the data and not to prove or disapprove hypotheses or to test a previous finding, thus we did not use themes from headlines in the iCBT program. The latent approach involves interpretative work and admits exploration of underlying ideas and assumptions, and conceptualizations in order to find themes with a broader meaning in the data [22].

.The analyses followed the six phases of thematic analysis described by Braun and Clark [22]. In the first phase, all transcribed data were read through to obtain an overall sense of the content and to note initial ideas. In the second phase, coding was performed to reduce the amount of data and to perform a more conceptual reading of the transcriptions in relation to the research question. In phase three, a search to define early themes was performed. In phase four, the themes were sorted into broader and meaningful themes by looking for recurring patterns. In phase five, the themes were then sorted into larger themes, and finally defined and named. The relationships between main themes and sub-themes were established to reflect upon the research question before finally in phase six, producing an initial report.

To ensure the credibility, triangulation through multiple channels was conducted by having more than one researcher independently analyzing the same data set and thereby considering selective perceptions and interpretive bias [30]. The triangulation was performed in three steps. In the first step the first author and four of the co-authors (MW, GM, MN, JL, PJ) independently performed the first steps of the thematic analysis, analyzing five randomly chosen transcribed interviews. In this step, early themes were tentatively defined. The early themes were then compared among the authors for selective perceptions and the authors then agreed upon the initial themes. In the second step, another five transcripts were analyzed and coded by the first author and three co-authors (MW, GM, JL, PJ). The remaining 10 transcripts were analyzed by the main author (MW). The authors then compared the coding in an iterative process and the themes were reviewed. In the third and last step, all co-authors discussed, revised, defined, and named the final themes (see coding schedule Table 3). The themes and alternative explanations of the results, if they occurred, were tested during the initial and final analysis phases. During the analysis of the final interviews no new themes emerged, supporting the belief that the maximal variation in the purposive sample had been reached. With permission from the patients the telephone interviews were tape-recorded and transcribed verbatim for data analysis, which increased trustworthiness. Apart from triangulation, trustworthiness was established by transparency of the quotations and the audit trail of the analysis process.

**Table 3:** Coding schedule

Meaning unit	Code	Category	Theme
*“Realizing that it’s up to me how I deal with life. Perhaps you can get that out of such a program. Not believing that the healthcare service should help me with everything. I’m not a child who needs their mom and dad to help, I’m an adult and I have to take charge of my life, and it’s up to me what I make of it.”* *“I really… that I made the effort to do those things that I wanted to do… that I know, that I knew before that it was good, but that I didn’t do it anyway for various reasons”* *“Really see yourself from outside, and how you feel and so on. You’re open in some way, and, like, want an improvement”*	Being driven to change Working towards change Interest in their health Internal motivation External motivation	Being one’s own guide	**Taking control of the disease**
*“You had that realization, and that you sometimes plan afterwards not to take on too many things and so one thing at a time”* *“Should it be like this? Shouldn’t there be more demands? And so, I gradually came to understand that that was the point”* *” Because there’s a fear linked to this issue of the heart not behaving. But the fear disappeared, and I learnt that the heart can cope with much more than you think”*	Changed thinking New knowledge as part of improving health Using the program to help control the disease	New insights	
*“So, the realization I had that it was OK to change my strategy, to change my personal strategy.”* *“I also felt in some way that what I was trying to convey, that came out in the e-mail. I got feedback on what I wanted”* *“I wrote based on how I felt. Yes, now there was, like, no time limit”* *“The advantage here was that you worked with the program during this time”* *“And there are painful things that affect me deeply, so in any case I sit there in peace and quiet, and I can go away if I can’t continue”*	Experienced freedom working in the program Flexibility in program motivates	Freedom	
*“Yes, it’s more… cognitive behavioral therapy is more work, but it also produces results”* *“Sometimes maybe you felt that, shit, perhaps I haven’t done as much as I should have with this homework this week”* *“I have what they call procrastination behavior, and that meant that I felt I was always slightly behind. And that I then got reminders, and it was good that I got reminders, because I needed that kick, as it were”*	Experiences requirements in the program as difficult The program is perceived as demanding Requires your own work effort	Demanding work	**Not just a walk in the park**
*“You try to repress things sometimes, unfortunately. Yes, it’s both good and bad, but eventually it re-emerges then, but no, I think what’s worth most is that you have to, err… think about it and above all look forward. That’s what has helped most, that there’s a change for the better in everything, both physically and mentally. That’s what I think has been sensible.”* *“Yes, then I cried and had… because there were questions that I just felt, ow, now you’re pushing with all your might, and then I started crying. That was sensitive”* *“There was a lot of these, that you were forced to reflect on things that you didn’t have. That you’d buried a bit”* *“Too often, perhaps, people feel so excluded and lonely with those worries they have in connection with these things”*	Uncertainty Anxiety associated with heart disease Feeling betrayed	Evoking emotions	
*“I perceived it almost as if perhaps it wasn’t designed for a… someone who works, like, and has a lot of different types of activities”* *“It meant that these final modules, where you had to weigh up your various activities over the days, for example, they became a little hard to deal with, I would say. I gave up a little bit there. It became a bit complex to sit down and do it”*	Wrong perspective on the disease Uncertainty about the content of the program	Not my cup of tea	
*“You can communicate with someone about the problems that you might recognize”* *“And it can be good to have the chance to express your feelings and so on, even if it’s online in the form of… You still get an answer from somewhere”*	Getting to communicate with someone about how you feel Getting to express their feelings Being heard	Being seen	**Being recognized and having support**
*“I saw other ways to behave, that I made sure that I went out, in particular. Not sitting at home all the time. I signed up for a reading group, which I’m still actually a member of. And then I signed up voluntarily, yes, voluntarily of course, for a new gymnastics troop”* *“I think a lot of it was the insight that, yes several insights actually not to set goals that are too high.”*	Organizing a health plan as a contribution to improvement Clear goals as a contribution to improvement	Support and structure	
*“The direct contact you can have with a treatment provider or a therapist. And what I found absolutely wonderful, what… perhaps now I’m saying something that, I don’t know if I can say it, but I found what… what gave me a completely different… expectation or understanding”* *“I really think that’s a criterion for someone who can understand the patient who is sitting on the other end of the computer”*	Strong alliance with the therapist The therapist as a listener Understanding of the patient Perceived as close and personal	The therapist was an important support	

## Results

Data from 20 patients, 11 men and 9 women, ranging in age from 34 to 79 years with an average age of 62 and most of the patients had a university degree *n* = 11 (55%). The patients had completed five to seven modules out of seven possible modules included in the 9-week iCBT program. The patients lived in both rural and urban areas, and most of the patients (*n* = 17) were in a relationship (Table 1).

**Fig. 1:**
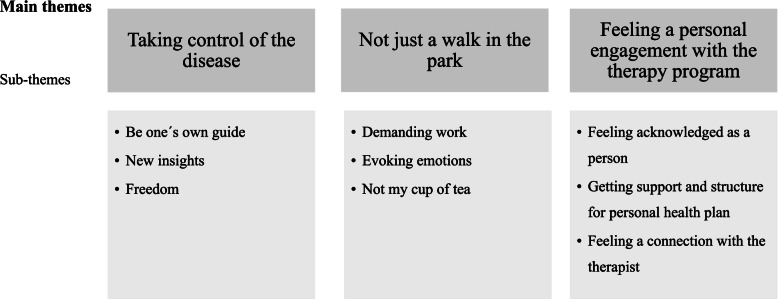
Main themes and sub-themes describing experiences of patients with cardiovascular disease and depression with Internet-based cognitive behavioral therapy

Three main identified themes were (a) Taking control of the disease, (b) Not just a walk in the park, and (c) Feeling a personal engagement with the therapy program. Each of these three main themes has three corresponding sub-themes. Themes and sub-themes are presented in, Fig. 1.

### Taking control of the disease

This first main theme describes how the patients experience taking control of their health by working in the program. The patients described how the program gave them guidance through the different modules, which made them feel they were part of the treatment process. They also felt that they could make their own decisions in their treatment based on the new knowledge they had obtained from the program about their disease. The patients experienced a sense of control over the treatment due to the perceived freedom of being able to go back and forth in the program and work at their own pace.

#### Be one’s own guide

The patients noted that being involved in the treatment and making their own decisions in the program created a feeling of being in control. The feeling of being in control made it easier to take difficult steps forward in order to improve their health by changing their negative thinking and behavior.

*“Realizing that it’s up to me how I deal with life. Perhaps you can get that out of such a program. Not believing that the healthcare service should help me with everything. I’m not a child who needs their mom and dad to help, I’m an adult and I have to take charge of my life, and it’s up to me what I make of it” participant no.14 Female.*

#### New insights

The patients reported that learning about their disease helped them gain new insights. Working with the program was helpful to gain a new understanding of how to live with CVD and depression. This also helped them perceive their situation as less stressful and made it easier for them to make decisions in the program concerning the necessary steps to change their negative emotions, thoughts, and behaviors.

*“Because there’s a fear linked to this issue of the heart not behaving. But the fear disappeared, and I learnt that the heart can cope with much more than you think. And I also learnt that this little leakage I have, that’s quite normal in old age” participant no.11 Female.*

#### Freedom

The patients experienced a feeling of freedom regarding the program. Having the possibility to influence the date and time to work with the program was perceived as flexible, but above all it contributed to a feeling that the program was constantly present during the whole treatment period and did not take breaks between the individual treatment sessions. The experienced freedom motivated the patients to continuously work with the treatment to achieve changes and take control of their own health.

*“One advantage… In other words, in a physical meeting there is… It’s perhaps that you have a physical meeting today, and then you meet again in two weeks. The advantage here was that you worked with the program during this time” participant no.10 Male.*

### Not just a walk in the park

In this second main theme, the patients describe the work in the program as demanding and emotionally challenging but necessary to achieve a change in their negative emotions, thoughts, and behaviors. Not all the patients participating in the program were satisfied with the program and some of the content was experienced as demanding and difficult.

#### Demanding work

The program was in some parts perceived as demanding and the patients had to work harder than they expected. For some patients, the amount of work and the active participation needed made it hard to complete the treatment despite experiencing positive changes in their negative thinking.

*“You have to read through, you have to reflect, you have to work for yourself. But you know that you’ll get an answer. Yes, it’s more… cognitive behavioral therapy is more work, but it also produces results” participant no.13 Male.*

#### Evoking emotions

Working on the program evoked emotions and led to feelings of discomfort and stress. These emotions were a result of the increased insights about their health and the changes needed to achieve more positive thinking and behavior. Patients expressed these evoked emotions as a necessary part of the process towards improvement and recovery from depression.

*“You try to repress things sometimes, unfortunately. Yes, it’s both good and bad, but eventually it re-emerges then, but no, I think what’s worth most is that you have to, err… think about it and above all look forward. That’s what has helped most, that there’s a change for the better in everything, both physically and mentally” participant no.3 Male.*

#### Not my cup of tea

Not all the patients were satisfied with the program. The text in the program was sometimes experienced as difficult to read and to recognize, and not to be tailored to their own situation This made them find the program less credible and thereby less helpful.

*“I perceived it almost as if perhaps it wasn’t designed for a… someone who works, like, and has a lot of different types of activities. It became very complex, filling in these weekly schedules based on the module” participant no.17 Male.*

### Feeling a personal engagement with the therapy program

In the third main theme, patients emphasized the importance of recognizing themselves in the program and relating the content of the program to their CVD and depression. The patients perceived the work structure in the program, including support and feedback from a trusted therapist, as an important factor in achieving changes in emotions, thoughts and behaviors.

#### Feeling acknowledged as a person

The patients felt that the tailored content of the program was personally directed towards patients with CVD and depression, creating a sense of being seen and listened to. Being able to relate the content of the program to their CVD and depression gave a feeling of safety and a realization of not being alone in their disease. This helped them to express their emotions and thoughts more openly about their disease, which in turn contributed to behavior changes, and thus improvements in their depression.

*“Sometimes you talk about depression and then if you don’t recognize it, so perhaps you don’t read that much, but if you recognize yourself there then you look up why you’re depressed and what you can do” participant no.1 Male.*

*“And it can be good to have the chance to express your feelings and so on, even if it’s online in the form of… You still get an answer from somewhere. I think that can be a positive thing. Just being able to express yourself can be enough” Participant no.4 Male.*

#### Getting support and structure for a personal health plan

Patients reported that the structure of the program helped them set individual goals, organize, and perform activities, which was experienced as helpful in order to achieve changes in emotions, thoughts, and behaviors. For example, the psychoeducation regarding the cardiovascular disease, and creating an individual activity plan, were experienced as important support in order to break the vicious circle including negative emotions, thoughts, and behaviors. New insights motivated them to increase their physical and social activities and learn to accept their cardiovascular disease.

*“Ideas about how to activate yourself, too. Like, planning your day better. And that one thing leads to the other, as it were. That you, I mean, just by activating yourself it’s easier to get out of a vicious circle, in a manner of speaking” participant no.12 Male.*

#### Feeling a connection with the therapist

The written feedback was experienced as trustworthy and made the patients perceive the therapist as credible, which also facilitated changes in emotions, thoughts, and behaviors. For most patients, the therapist was perceived as a close personal guide throughout the program, who made it possible to complete those parts of the program that were seen as difficult. The therapist also helped them become aware of and reflect upon, and thus change their negative emotions, thoughts, and behaviors.

*“Otherwise, I think that the feedback has been adequate, and I’m sometimes surprised how much the person providing the feedback has understood about what I’ve actually thought, when I’ve done my exercises. So, it’s been good” participant no.17 Male.*

*“I felt that I was treated very personally then, and it was a bit like sitting next to the person. There was very close, direct contact” participant no.11 Female.*

## Discussion

The aim of this study was to explore CVD patients’ experiences of engaging in a tailored iCBT program. We found three main themes “Taking control of the disease”, “Not just a walk in the park”, and “Feeling a personal engagement with the therapy program”.

An important finding was that the program helped the patients to change their emotions, thoughts, and behaviors, by learning to take control of the disease and treatment and learning to live with cardiovascular disease. This is in line with previous research [31, 32] investigating the effects of iCBT in depressed patients without cardiovascular disease. These studies showed that patients who are active and take responsibility for their treatment attribute improvements from the treatment to themselves and the program thus produces a strongly favorable outcome. This can be seen as taking control over and improving one’s own situation. A previous study has shown that cardiovascular patients with depression and/or anxiety want to go back to normal but lack the ability to take control over their lives after a cardiac event [33]. In our study, control was characterized by being able to go beyond self-constructed limitations related to cardiovascular disease. For example, in our and other studies patients described having negative thoughts and avoiding participation in social or physical activities due to fear and worries related to their cardiovascular disease. Thus, this may lead to a restricted life, which in turn can cause negative perceptions of living with cardiovascular disease [33, 34], thereby causing a vicious circle. Our findings suggest that participation in iCBT can help depressed cardiovascular patients go beyond their self-constructed limitations, possibly increasing their confidence so that they are capable of changing their emotions, thoughts, and behaviors and thus breaking the vicious circle and taking control over their cardiovascular disease.

Another finding was the experience that participation in iCBT requires a great deal of effort to achieve positive changes in emotions, thoughts, and behaviors. The patients perceived the work in the program as stressful with regard to the need to read text and perform homework assignments according to a time schedule. They also described the program as challenging since they had to reevaluate how they were feeling, thinking, and behaving in relation to their CVD and depression. However, patients described the work in the program also as rewarding since the challenges made them achieve positive changes. This confirms that it is important that such programs require active engagement from the patients and that their beliefs are challenged in order to change emotions, thoughts, and behaviors [32]. However, it is important to keep in mind that working with the iCBT program can also cause stress and discomfort [17, 21, 35, 36], and thus is “not just a walk in the park”. In spite of this, in our main RCT study a total of 60% of the participants completed the whole iCBT program, and 82% completed more than half of the program [19]. This suggests that iCBT can be challenging and may not fit all, although our [19] and other studies have shown that it can fit the vast majority of patients [37].

.In one of the findings the patients describe a feeling of a personal engagement with the iCBT program. The patients stated that the work structure – including the tailored content, the homework assignments, and the feedback from the nurse therapist – helped them feel that they were being seen and supported in order to achieve positive changes in emotions, thoughts, and behaviors.

The tailored content of the program helped the patient to create a feeling of recognition and being acknowledged as a person, thus making it easier to accept and work toward positive changes. In previous research, patients describe a desire for more disease-specific content related to the somatic chronic disease [15, 16]. The patients in our study described being able to relate to the content of the program, which led to positive changes in emotions, thoughts, and behaviors. This suggests that it is important that the content of the iCBT programs is tailored to the specific disease to help patients recognize themselves and contribute to a favorable outcome and adherence to the program. The patients also reported that the program’s weekly timeframe, including disease-oriented education and assignments, gave structure and support. A previous study by Hermes at al [38]. reported that the demanding structure and strict timeframe could be experienced as barriers and lead to attrition. This can be seen as contradictory, although in our study patients stated that the assignments helped them set goals and become more actively involved in their social lives, even when the schedule and timeframe was demanding. This finding is supported by other studies where patients were positive about being confronted with the challenge of a timeframe in their CBT programs [32, 39]. Hence, iCBT programs are generally characterized by clear contents, structures and timeframes. However, this does not preclude such programs at the same time being designed to be perceived as permissive and free, for example as described in the sub-theme freedom, in order to fit as many patients as possible.

An important part of feeling a personal engagement with the therapy program seemed to be the feedback in the program, which was described as motivating to achieve positive changes in emotions, thoughts, and behaviors. The importance of a therapist giving feedback has been established in previous studies and is an important factor in achieving good treatment results from iCBT [21, 31, 39, 40]. This can be related to the establishment of a therapeutic alliance, which is an important part of CBT in order to achieve positive treatment results [41]. In our study, the therapists had knowledge of both CVD and depression, which could explain why the therapist was seen as an experienced healthcare professional and a reliable guide in the program. In addition, the therapist was also perceived as a good listener who helped them set achievable treatment goals and construct realistic plans, which helped them create realistic expectations of how to live with their disease. This indicates to establish a therapeutic alliance when delivering iCBT to patients with a chronic somatic disease such as CVD combined with depression, it is important not only to have a therapist, but also that the therapist should have knowledge of the specific disease as well as knowledge of depression. In our study we used nurses with only brief introductory training in CBT as therapists, but other healthcare professionals with disease-specific knowledge can also learn to deliver iCBT with good treatment results [14, 42].

### Strengths and limitations

The strength of this study is that it is one of the first to report on patients’ experiences of participating in an iCBT program tailored to patients with CVD, which contributes to a better understanding of patients’ preferences when developing future programs.

However, our findings should be interpreted with some caution. First, we sought to investigate experiences of a specific treatment using existing theories and therefore estimated the number of participants to be enough to contribute substantially to the understandings [43]. The characteristics of the participants in this interview study did not differ compared to the total population in the RCT study. The average age of the participants in this interview study (62 years) was comparable to other studies that have included CVD patients with depression and who report an average age of 61 and 63 years respectively [3, 44] Regarding adherence in the RCT study [19] 82% of the participants completed more than half of the modules in the iCBT program. This is similar to other iCBT studies CVD [23, 45] reporting an adherence of 92 and 60% respectively completed more than half of the treatment program. Over half of the patients in this study had a university degree which is higher in comparison to similar iCBT studies [45, 46] and slightly higher in comparison of degree of education to the general population in Sweden. Also, we used a strategic sample, and therefore cannot claim that the persons included are representative of the whole population of CVD patients with depression or patients with depression or even of those who were included in the original trial.

We invited all the patients who had participated in spring 2017. However, those who agreed to participate in the interview study had completed at least four modules of the iCBT program. This is a limitation, as those who agreed to participate may have been more positive about the program. We did not measure depression severity when inviting the participants to this qualitative study.

Another limitation is that the interviews were conducted 1 to 6 months after completion of the iCBT program. The reason for this was due to time constraints. This may have influenced the content of the responses due to difficulty remembering specific details. However, when the interviews were conducted, it never occurred that patients had to struggle to remember. On the contrary, the patients seemed to have a clear picture from the time in the program. When analyzing the interviews, the content did not seem to vary depending on when these were conducted.

All the interviews were conducted by telephone, which was deemed appropriate for collecting the data. The procedure may, however, have affected the depth of the content of the interviews compared with conducting face-to-face interviews. Telephone interviewing has been reported as equally valid compared to face-to-face interviewing for data collection [47] and has also been found to be well accepted by participants in interview studies and not to affect the final findings [47, 48].

## Conclusions

Engaging in an iCBT program tailored for patients with CVD and depression was by the patients perceived as helpful in the treatment of depression. They experienced positive changes in emotions, thoughts, and behaviors. This was facilitated by learning to take control of their CVD, being confirmed and getting support. The patients described that the therapist enabled a positive change by guiding them through the program, including both psychosocial and somatic aspects associated with CVD and depression. The patients also experienced the work in the program as demanding and emotionally challenging. However, this was necessary to achieve changes in emotions, thoughts, and behaviors. Future improvements in iCBT for CVD patients with depression could be to make the program more person-centered by allowing them to tailor the content of the program to their own preferences.

## Supplementary Information

**Additional file 1.**

## Data Availability

The data that support the findings of this study cannot be made publicly available for confidentiality reasons. Data are however available from the corresponding author upon reasonable request.

## Abbreviations

CBT: Cognitive behavioral therapy
CVD: Cardiovascular disease
iCBT: Internet-based cognitive behavioral therapy
PHQ-9: Patient Health Questionnaire 9
