# Supplementary material for: The experience of participating in an internet-based cognitive behavioral therapy program among patients with cardiovascular disease and depression: a qualitative interview study
Source: BMC Psychiatry. 2022 Apr 25;22:294. doi: 10.1186/s12888-022-03939-7 (PMC9036745; doi:10.1186/s12888-022-03939-7)
Supplement: Supplementary file 1 — Additional file 1. [file 12888_2022_3939_MOESM1_ESM.pptx]

## Slide 1
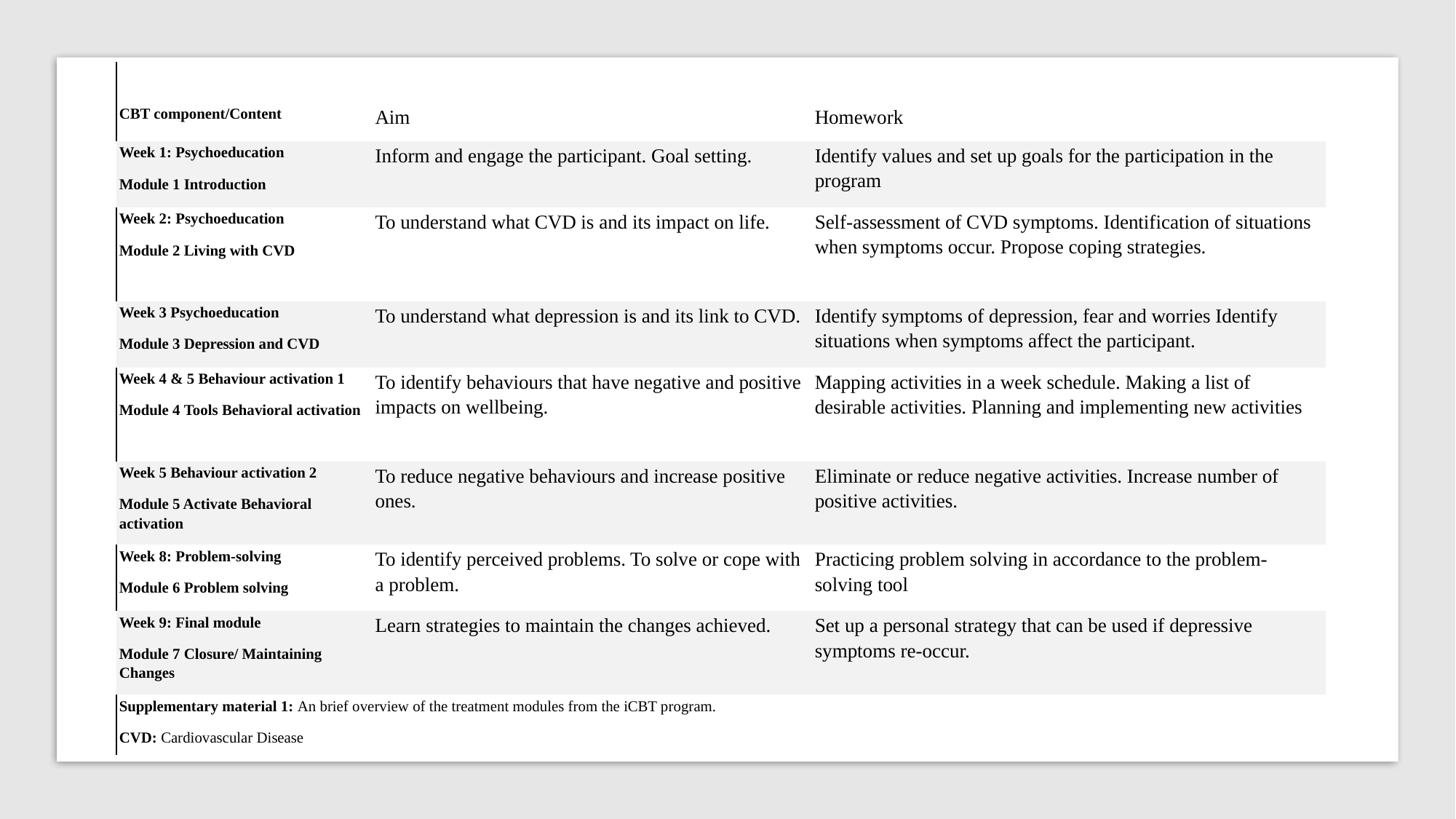

| | | |
| --- | --- | --- |
| CBT component/Content | Aim | Homework |
| Week 1: Psychoeducation Module 1 Introduction | Inform and engage the participant. Goal setting. | Identify values and set up goals for the participation in the program |
| Week 2: Psychoeducation Module 2 Living with CVD | To understand what CVD is and its impact on life. | Self-assessment of CVD symptoms. Identification of situations when symptoms occur. Propose coping strategies. |
| Week 3 Psychoeducation Module 3 Depression and CVD | To understand what depression is and its link to CVD. | Identify symptoms of depression, fear and worries Identify situations when symptoms affect the participant. |
| Week 4 & 5 Behaviour activation 1 Module 4 Tools Behavioral activation | To identify behaviours that have negative and positive impacts on wellbeing. | Mapping activities in a week schedule. Making a list of desirable activities. Planning and implementing new activities |
| Week 5 Behaviour activation 2 Module 5 Activate Behavioral activation | To reduce negative behaviours and increase positive ones. | Eliminate or reduce negative activities. Increase number of positive activities. |
| Week 8: Problem-solving Module 6 Problem solving | To identify perceived problems. To solve or cope with a problem. | Practicing problem solving in accordance to the problem-solving tool |
| Week 9: Final module Module 7 Closure/ Maintaining Changes | Learn strategies to maintain the changes achieved. | Set up a personal strategy that can be used if depressive symptoms re-occur. |
| Supplementary material 1: An brief overview of the treatment modules from the iCBT program. CVD: Cardiovascular Disease | | |
